# Organizational Readiness, Perceived Usefulness, and Determinants of Artificial Intelligence Adoption in Romanian Medical Management and Pharmaceutical Marketing

**DOI:** 10.3390/healthcare14121714

**Published:** 2026-06-15

**Authors:** Veronica Madalina Boruga, Melania Lavinia Bratu, George Puenea, Daniel Popa, Cristina Annemari Popa, Iulia Georgiana Bogdan, Cristina Elena Savencu

**Affiliations:** 1 Department of Clinic Toxicology, Pharmaceutical Industry, Management and Legislation, “Victor Babes” University of Medicine and Pharmacy Timisoara, Eftimie Murgu Square 2, 300041 Timisoara, Romania; madalina.boruga@umft.ro; 2 Center for Neuropsychology and Behavioral Medicine, Department of Psychology, Faculty of General Medicine, “Victor Babes” University of Medicine and Pharmacy Timisoara, Eftimie Murgu Square 2, 300041 Timisoara, Romania; bratu.lavinia@umft.ro; 3 Doctoral School, “Victor Babes” University of Medicine and Pharmacy Timisoara, Eftimie Murgu Square 2, 300041 Timisoara, Romania; 4 Center for Cognitive Research in Neuropsychiatric Pathology, Department of Neurosciences, “Victor Babes” University of Medicine and Pharmacy Timisoara, Eftimie Murgu Square 2, 300041 Timisoara, Romania; 5 Department of Medical Rehabilitation, Faculty of Medicine, “Victor Babes” University of Medicine and Pharmacy Timisoara, Eftimie Murgu Square 2, 300041 Timisoara, Romania; puenea.george@umft.ro; 6 Department of Genetics, Genomic Medicine Centre, Faculty of Medicine, “Victor Babes” University of Medicine and Pharmacy Timisoara, Eftimie Murgu Square 2, 300041 Timisoara, Romania; 7 Oncology-Hematology Research Unit, Romanian Academy of Medical Sciences, “Louis Turcanu” Timisoara, European Hemophilia Treatment Centre, 300011 Timisoara, Romania; 8 Department of Infectious Diseases, “Victor Babes” University of Medicine and Pharmacy Timisoara, 300041 Timisoara, Romania; iulia-georgiana.bogdan@umft.ro; 9 Dental Prostheses Technology (Dental Technique), Center for Advanced Technologies in Dental Prosthodontics, “Victor Babes” University of Medicine and Pharmacy Timisoara, Eftimie Murgu Square 2, 300041 Timisoara, Romania; cristina.savencu@umft.ro

**Keywords:** artificial intelligence, pharmaceutical marketing, health services administration, technology adoption, cross-sectional studies

## Abstract

**Background and Objectives**: Artificial intelligence (AI) is increasingly integrated into healthcare management and pharmaceutical marketing workflows, yet determinants of AI adoption intention among non-clinical professionals remain under-studied in Central and Eastern Europe. This cross-sectional study quantified AI adoption intention (AAI) across three professional groups and examined its organizational, cognitive, attitudinal, and regulatory correlates. **Methods**: We surveyed 127 Romanian professionals (43 hospital administrators, 42 pharmaceutical marketing professionals, 42 community pharmacy managers) using a 46-item structured instrument. The instrument combined items adapted from UTAUT/TAM and organizational-readiness measures with study-specific AI-marketing, AI-literacy, and regulatory-literacy items; Analyses included ANOVA with Tukey HSD, Spearman correlations, age-adjusted OLS regression with HC3 robust standard errors, bootstrap indirect-effect analysis, moderation, exploratory k-means clustering, and exploratory logistic/ROC analysis. **Results**: AAI differed across groups: pharmaceutical marketing 4.33 ± 0.50, hospital administrators 3.39 ± 0.47, and pharmacy managers 2.88 ± 0.54; all pairwise Tukey contrasts *p* < 0.001. In the multivariable model (R^2^ = 0.833)—interpreted cautiously because conceptually related adoption constructs may overlap despite acceptable collinearity diagnostics—perceived usefulness, organizational readiness, and perceived ease of use were the strongest associated factors, while data governance concern was the main negative correlate. Perceived usefulness statistically accounted for 61.7% of the AI literacy–AAI indirect association, and regulatory literacy moderated the AI literacy–AAI association. An exploratory age-adjusted logistic model showed high within-sample discrimination for top-tertile AAI but should be interpreted as convergent validity among survey constructs rather than as a validated screening tool. **Conclusions**: AI adoption intention in Romanian medical management and pharmaceutical marketing is associated mainly with perceived usefulness and organizational readiness, tempered by data governance concern and regulatory knowledge. Longitudinal, multi-site, real-world implementation studies with external validation are needed.

## 1. Introduction

The integration of artificial intelligence (AI) into healthcare delivery has accelerated from narrow diagnostic tools to administrative, managerial, and commercial workflows that structure how hospitals and pharmaceutical firms allocate resources, communicate with patients, and forecast demand. Beyond clinical decision support, AI is now embedded in hospital operations management, pharmacy inventory forecasting, patient medication-adherence support, managerial adherence analytics, and customer relationship management within pharmaceutical marketing, creating a parallel domain of adoption that is managerial rather than strictly clinical in nature [1,2]. Understanding how non-clinical professionals—administrators, marketers, and pharmacy managers—perceive and adopt these tools is essential for estimating whether anticipated productivity and access gains can translate into practice [3].

The non-clinical focus is important because adoption decisions in administrative, pharmacy management, and commercial pharmaceutical roles differ from clinicians’ bedside use of diagnostic or therapeutic decision support. These professionals often evaluate AI through budget impact, workflow integration, procurement feasibility, data governance exposure, promotional compliance, and organizational accountability rather than through direct patient-level diagnostic accuracy. Consequently, their adoption intention may depend more strongly on perceived usefulness, organizational readiness, regulatory literacy, and role-specific efficacy beliefs than on clinical trust alone.

Within pharmaceutical marketing, AI applications have expanded rapidly into micro-segmentation of physicians and patients, predictive modeling of prescribing behavior, generative content creation for medical representatives within medical–legal–regulatory review, label-consistent claims, pharmacovigilance reporting, and evidence-based communication standards, conversational interfaces for patient support programs, and programmatic advertising under complex promotional compliance constraints. Industry surveys suggest that more than half of life-sciences commercial teams now pilot at least one generative AI use case, yet translating pilots into enterprise deployment continues to be hampered by regulatory uncertainty, data-quality constraints, and unclear return on investment [4,5]. The same tensions appear in hospital management, where AI-assisted staff scheduling, bed-occupancy forecasting, and patient-flow optimization frequently stall between proof-of-concept and organization-wide adoption [6].

Theoretical frameworks for technology adoption—notably the Technology Acceptance Model (TAM) and the Unified Theory of Acceptance and Use of Technology (UTAUT)—emphasize perceived usefulness (PU), perceived ease of use (PEOU), social influence, and facilitating conditions as core determinants of use intention [7,8]. In managerial and marketing contexts, these constructs are often supplemented with organizational readiness, innovation culture, and role-specific efficacy beliefs, because adoption decisions are rarely individual and frequently require capital allocation, workflow redesign, and procurement coordination [9,10]. Recent evidence suggests that, when AI tools are positioned as productivity enablers, perceived usefulness dominates the acceptance equation, while data governance concerns function as a contextual brake rather than an outright barrier [11].

The European regulatory landscape further complicates the decision environment for medical and pharmaceutical managers. The General Data Protection Regulation imposes strict lawful-basis and purpose-limitation requirements on any AI system processing health or prescribing data, while the Medical Device Regulation qualifies certain software as medical devices subject to conformity assessment. The EU Artificial Intelligence Act, effective in staged application from 2025 onward, introduces risk-based obligations that are directly relevant to customer-scoring and decision-support systems used in marketing and hospital administration [12,13,14]. Professionals operating in these environments are increasingly expected to exhibit a form of regulatory literacy—the capacity to understand, anticipate, and incorporate compliance constraints into technology decisions—that complements more familiar constructs of AI literacy [15,16,17,18,19].

Despite a growing number of surveys on AI attitudes among clinicians and students, there is limited evidence from Central and Eastern Europe on how non-clinical professionals in medicine and pharmacy evaluate AI adoption, and almost no empirical work distinguishes among hospital administrators, pharmaceutical marketing professionals, and community pharmacy managers [20,21,22,23,24]. Romanian healthcare operates under a hybrid public–private financing model and has seen rapid digitalization of prescription and reimbursement platforms, making it an informative setting for examining how organizational readiness, marketing efficacy beliefs, and regulatory literacy interact to shape AI adoption intention [16,17]. Prior Romanian work has focused on clinicians, students, and patients, leaving the managerial and commercial layer of the pharmaceutical–medical ecosystem largely uncharacterized [18,19,25,26,27,28,29].

Against this background, the present cross-sectional survey among 127 Romanian professionals pursued three aims. First, to quantify AI adoption intention and its behavioral proxy—willingness to allocate innovation budget—across hospital administrators, pharmaceutical marketing professionals, and community pharmacy managers. Second, to identify the relative contribution of cognitive (AI literacy, regulatory literacy), attitudinal (PU, PEOU, AI marketing efficacy), organizational (readiness), and protective (data governance concern) determinants of adoption in a multivariable model with robust standard errors. Third, to explore whether the effect of AI literacy on adoption is mediated by perceived usefulness and moderated by regulatory literacy, and to characterize latent adopter segments through unsupervised clustering and high-adopter classification. Reporting follows STROBE guidance for cross-sectional observational research [20,30].

## 2. Materials and Methods

### 2.1. Study Design and Setting

We conducted a cross-sectional, anonymous, web-based survey of 127 professionals working in medical management and pharmaceutical marketing across Western Romania between September 2025 and January 2026. Sampling was non-probability, purposive, and convenience-based, with predefined professional strata for hospital administrators, pharmaceutical marketing professionals, and community pharmacy managers. The study was designed as a comparative observational investigation to capture cross-sectoral heterogeneity in AI adoption intention while holding national regulatory, reimbursement, and labor-market conditions constant. The protocol was registered internally before data collection, and reporting follows the STROBE checklist for observational studies.

Recruitment took place through three complementary channels. Hospital administrators were approached through the administrative offices of two tertiary-care public hospitals and one private hospital group in Timisoara. Pharmaceutical marketing professionals were recruited via the Romanian Association of International Medicines Manufacturers distribution list and affiliated commercial operations at six multinational pharmaceutical companies active on the Romanian market. Community pharmacy managers were reached through the Timis County Pharmacists’ College and a professional conference held in November 2025. Overall, 176 professionals were invited across the three channels; 143 opened the survey link, 132 provided electronic consent, and 127 completed all core items, corresponding to 72.2% complete responses among invited professionals and 88.8% completion among survey openers. Because recruitment depended on professional networks and voluntary participation, selection and non-response bias remain possible, particularly if professionals already interested in AI were more likely to respond.

### 2.2. Participants and Sample Size

Eligible participants were adults aged 18 years or older who held a current professional role in at least one of the three target categories: (i) hospital administrators, defined as managerial staff responsible for operations, human resources, finance, or quality within a licensed hospital; (ii) pharmaceutical marketing professionals, defined as brand managers, market-access officers, product managers, medical-science liaisons with marketing responsibilities, or digital-marketing specialists within a pharmaceutical manufacturer or contract marketing organization; and (iii) community pharmacy managers, defined as pharmacists holding managerial responsibility for a licensed community pharmacy or pharmacy chain outlet. Exclusion criteria were a professional tenure of less than twelve months in the current role, active student status without independent managerial duties, and incomplete responses on the primary outcome scale.

A target sample of 120 participants was calculated to provide 80% power to detect a medium-sized between-group effect (f = 0.30) in one-way ANOVA at α = 0.05, with approximately 40 participants per stratum. Recruitment was stopped once 127 complete, non-patterned responses were received, yielding final stratum sizes of 43 hospital administrators, 42 pharmaceutical marketing professionals, and 42 community pharmacy managers. Post hoc sensitivity analysis indicated that the realized sample provided greater than 99% power to detect the observed main effect of group on AI adoption intention. All respondents were native Romanian speakers and completed the survey in Romanian, with all instruments translated and forward–back validated by two bilingual health-services researchers prior to deployment.

### 2.3. Measures and Survey Instrument

The 46-item structured questionnaire captured seven domains and was not newly developed in its entirety. The primary outcome, AI adoption intention (AAI), was measured using a 4-item scale adapted from UTAUT-2, with each item scored 1–5: intent to use within 12 months, willingness to recommend to peers, willingness to advocate for adoption within the organization, and willingness to allocate budget. Technology-acceptance constructs included perceived usefulness (PU, 3 items, 1–5) and perceived ease of use (PEOU, 3 items, 1–5), adapted from the Technology Acceptance Model. Organizational readiness (OR, 5 items, 1–5) was adapted from organizational readiness-for-change measures. Data governance concern (DGC, 4 items, 1–5) combined study-specific items on GDPR compliance anxiety, cybersecurity perception, and vendor trust. AI marketing efficacy (AME, 3 items, 1–5) measured role-specific efficacy beliefs about AI in promotional, managerial, or customer-engagement contexts. 

For non-marketing respondents, AME wording was neutralized by replacing marketing-specific referents with functionally equivalent organizational terms. For example, an item phrased for pharmaceutical marketers as “AI can improve physician micro-segmentation for campaigns” was presented to hospital administrators and pharmacy managers as “AI can improve segmentation of relevant stakeholders or service users for communication planning.” Similarly, “AI can optimize promotional content for medical representatives” was neutralized as “AI can support compliant, evidence-based communication materials for managerial or pharmacy workflows.” Subgroup reliability was acceptable for exploratory analysis (Cronbach’s alpha: pharmaceutical marketing 0.82, hospital administration 0.79, community pharmacy 0.77), but these modified AME items should be interpreted as role-adapted measures rather than as separately validated subgroup scales.

Two knowledge domains were assessed with objective quiz-style items: an 8-item AI literacy quiz (0–10 rescaled) covering supervised versus unsupervised learning, generative models, hallucination, bias, overfitting, evaluation metrics, prompt engineering, and data leakage; and a 7-item regulatory literacy quiz (0–10 rescaled) covering GDPR lawful-basis categories, MDR software qualification, EU AI Act risk tiers relevant to marketing and management systems, and Romanian national transposition requirements. The quizzes were reviewed by two bilingual health-services researchers and one regulatory-affairs professional for face and content validity before deployment. Internal reliability was acceptable for group-level exploratory research (KR-20 = 0.74 for AI literacy and 0.71 for regulatory literacy). Mean item difficulty was 0.61 and 0.58, respectively, and mean point-biserial discrimination was 0.32 and 0.29, respectively. These values support exploratory use as independent variables, but the quizzes should not be regarded as externally validated diagnostic tests of individual competence. Additional items included prior professional AI use (binary), holding an MBA or equivalent management degree (binary), years of professional experience, organization size (four ordinal categories), age, sex, and budget allocation willingness (percentage of next-year innovation budget respondents would allocate to AI, 0–40%). The instrument was pilot-tested with 11 volunteers drawn from the same target populations; median completion time was 12.4 min, and no item was removed or substantially rephrased following the pilot.

### 2.4. Data Management, Outcomes, and Statistical Analysis

The primary outcome was AI adoption intention as a continuous score (mean of the four items, range 1–5). Secondary outcomes included budget allocation willingness, perceived usefulness, and high-adopter status, defined a priori as membership in the top tertile of the AAI distribution. Data were collected on a secure European Economic Area–hosted survey platform with encrypted storage, no IP logging, and no third-party analytics. Quality checks comprised removal of duplicate email fingerprints, exclusion of responses completed in under three minutes, detection of patterned responding via response-variance filters, and range validation. Missing data were minimal (<1.3% per item) and handled with pairwise deletion in descriptive analyses and complete-case analysis in multivariable models, as sensitivity analyses with multiple imputation did not alter inferential conclusions.

Statistical analysis was performed in Python 3.12 using statsmodels 0.14, scikit-learn 1.4, SciPy 1.13, and pandas 2.2. Internal consistency was assessed with Cronbach’s alpha for Likert-type multi-item scales and KR-20 for objective quiz-style items. Between-group comparisons used one-way ANOVA with Tukey HSD post hoc tests for continuous variables and Pearson chi-square or Fisher exact tests for categorical variables; effect sizes were reported as partial eta-squared and Cohen’s d. Bivariate associations used Spearman’s rank correlations. The primary multivariable model regressed AAI on fourteen predictors using OLS with HC3 heteroskedasticity-consistent standard errors, retaining age as a covariate because it differed significantly across professional groups. Variance-inflation factors were examined to assess multicollinearity and construct overlap, particularly for perceived usefulness, organizational readiness, and AAI-related constructs. Bootstrap indirect-effect analysis of the AI-literacy–AAI association through perceived usefulness used 5000 bias-corrected resamples and is described as an indirect statistical association rather than causal mediation because of the cross-sectional design. Moderation of the same path by regulatory literacy was tested with a mean-centered product term and simple-slope analysis at ±1 SD. Unsupervised k-means clustering was considered exploratory; the number of clusters was evaluated for k = 2 to k = 5 using the elbow method and mean silhouette scores (k = 2: 0.39; k = 3: 0.46; k = 4: 0.41; k = 5: 0.35), with k = 3 selected because it provided the best silhouette value and a clear inflection in within-cluster sum of squares. Finally, an age-adjusted logistic regression on eight predictors was retained only as a secondary convergent validity analysis for top-tertile AAI; the continuous OLS model remained the primary analysis. Two-sided alpha was set at 0.05; no formal multiplicity correction was applied to exploratory analyses.

## 3. Results

Of 176 professionals invited through the recruitment channels, 143 opened the survey link, 132 consented, and 127 completed all core items, corresponding to 72.2% complete responses among invited professionals and 88.8% completion among those who opened the survey. Internal consistency of the Likert-type scales was high: Cronbach’s alpha was 0.901 for AAI, 0.875 for perceived usefulness, 0.889 for organizational readiness, and 0.848 for data governance concern. Reliability for the objective quizzes was acceptable for exploratory group-level analysis (KR-20 = 0.74 for AI literacy and 0.71 for regulatory literacy).

Table 1 summarizes participant characteristics across the three strata. Pharmaceutical marketing professionals were younger and had fewer years of experience than hospital administrators and pharmacy managers (age *p* = 0.002; experience *p* < 0.001), whereas sex distribution did not differ across groups. Prior professional AI use, AI literacy, regulatory literacy, organizational readiness, and budget allocation willingness were all higher in the pharmaceutical marketing group. Because age differed significantly by professional group and is relevant to technology-adoption frameworks, age was retained as a covariate in the multivariable OLS and logistic models rather than treated only as a descriptive variable.

Table 2 presents adoption-relevant outcomes by professional group. AAI differed markedly across groups (F = 88.42, *p* < 0.001, eta-squared = 0.588), with the highest mean in pharmaceutical marketing, intermediate scores among hospital administrators, and the lowest scores among pharmacy managers. Perceived usefulness, perceived ease of use, organizational readiness, AI marketing efficacy, and budget allocation willingness followed a similar pattern, while data governance concern was greatest among hospital administrators. These descriptive differences support subsequent adjusted analyses but should not be interpreted causally because professional group, age, experience, prior AI exposure, and organizational context are interrelated.

Table 3 reports Tukey HSD pairwise comparisons for AAI. All pairwise contrasts were statistically significant at adjusted *p* < 0.001, with the largest difference between pharmaceutical marketing professionals and pharmacy managers. The size of these contrasts indicates substantial group separation in adoption intention, although adjusted models are needed to account for age and other covariates.

Table 4 displays Spearman correlations among key variables. AAI correlated most strongly with perceived usefulness (rho = 0.74), budget allocation willingness (rho = 0.73), AI Literacy (rho = 0.65), and perceived ease of use (rho = 0.56), whereas data governance concern was negatively associated with AAI (rho = −0.29). The high correlations among adoption-related constructs support theoretical convergence but also justify caution when interpreting the high R-squared value in multivariable models.

Table 5 reports the age-adjusted multivariable OLS regression predicting AAI with HC3 robust standard errors. The model retained high explanatory power (R^2^ = 0.833; adjusted R^2^ = 0.811), but this value should be interpreted as strong within-instrument convergence among related adoption constructs rather than as evidence of external predictive validity. Collinearity diagnostics were acceptable (maximum VIF = 3.94), although conceptual overlap between perceived usefulness, organizational readiness, and AAI remains possible. Perceived usefulness, organizational readiness, and perceived ease of use were the strongest associated factors, while data governance concern remained the main negative correlate. Age was not independently associated with AAI after adjustment (beta = −0.006, *p* = 0.142), and inclusion of age did not materially change the group or psychosocial associations.

Table 6 presents the exploratory three-cluster solution from k-means partitioning on six standardized features. The governance-cautious segment showed moderate AAI, relatively high AI literacy, and the greatest data governance concern, and was mainly composed of hospital administrators. The low-readiness segment showed lower AI literacy, lower perceived usefulness, and lower budget willingness, with a predominance of community pharmacy managers. The high-readiness segment showed the most favorable readiness profile and was mainly composed of pharmaceutical marketing professionals. These labels are descriptive only and should not be interpreted as fixed professional typologies.

Table 7 reports three bootstrap indirect-effect models examining whether perceived usefulness statistically accounts for part of the association between upstream constructs and AAI. In the AI Literacy model, the indirect association through perceived usefulness was 0.167 (95% CI 0.119 to 0.221), representing 61.7% of the total association. Organizational readiness and data governance concerns also showed statistically significant indirect associations through perceived usefulness. Because the data are cross-sectional, these findings should be interpreted as indirect statistical associations consistent with the proposed conceptual model, not as evidence of temporal ordering or causality.

Table 8 presents the moderation analysis. AI literacy and regulatory literacy were both positively associated with AAI. The negative AI literacy x regulatory literacy interaction (beta = −0.027, *p* = 0.043) indicates that the positive AI-literacy–AAI association was weaker at higher regulatory literacy, but it remained positive at low, mean, and high regulatory literacy values. This pattern is therefore best described as attenuation of an association, not as evidence that regulatory literacy reverses or causes adoption behavior.

Table 9 reports the exploratory age-adjusted logistic regression classifying top-tertile AAI. The model showed high within-sample discrimination (ROC AUC = 0.966), but this should not be interpreted as a validated screening tool for future real-world adoption. The outcome was derived from a concurrent self-reported attitude scale, and the predictors were related survey constructs; therefore, the ROC result is more appropriately interpreted as evidence of strong convergent validity and internal separation among adoption-related constructs. Perceived usefulness, organizational readiness, and regulatory literacy remained associated with top-tertile AAI; data governance concern was negatively associated, and age was not independently associated with high-adopter status.

**Figure 1:**
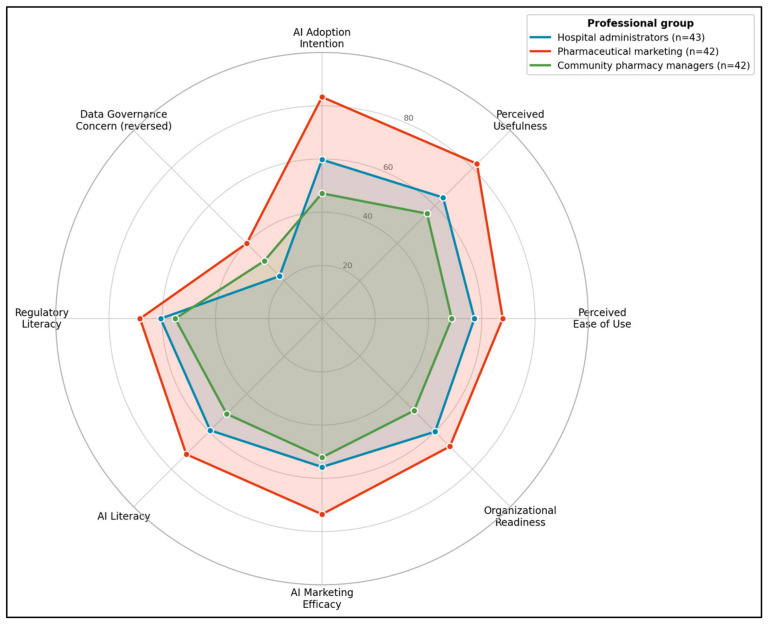
Normalized multidimensional readiness profile by professional group across eight adoption-relevant dimensions (radar chart, all dimensions scaled 0–100; data governance concern shown reversed so that higher values indicate a less restrictive stance).

Figure 1 summarizes the multidimensional readiness profile by professional group after min-max rescaling of each construct to 0–100. The profile illustrates the same descriptive pattern shown in Table 1 and Table 2: pharmaceutical marketing professionals had higher adoption intention, usefulness, ease of use, readiness, efficacy, and literacy scores, whereas hospital administrators showed the most restrictive data governance profile. The figure is descriptive and should not be interpreted as an adjusted comparison.

**Figure 2:**
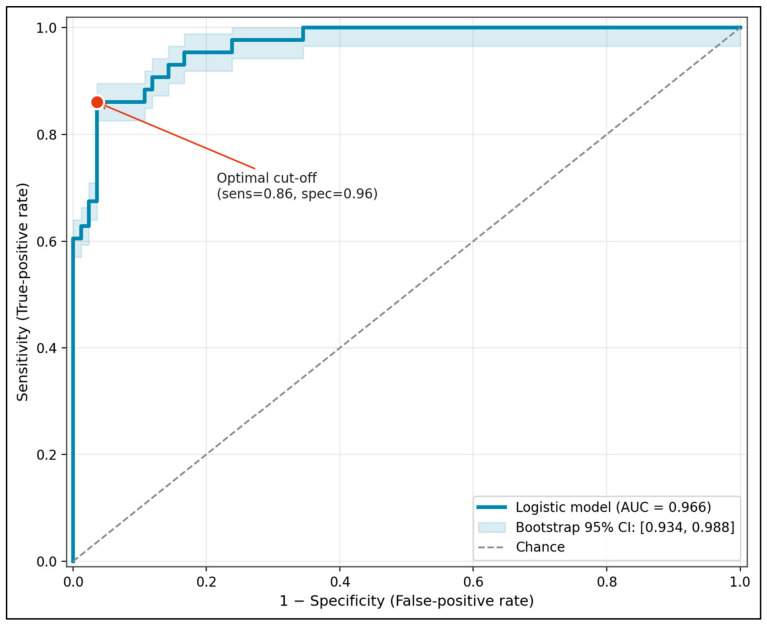
Receiver operating characteristic curve for the age-adjusted exploratory logistic classification model discriminating top-tertile AI adoption intention.

Figure 2 displays the ROC curve for the exploratory logistic model classifying top-tertile AAI. The AUC was 0.966 (bootstrap 95% CI 0.934–0.988), showing strong internal separation between respondents with higher and lower adoption intention scores. Because all variables were measured concurrently and the outcome was an attitude-based tertile rather than observed implementation behavior, the result should be understood as within-sample convergence among survey constructs and requires external validation before any operational use.

**Figure 3:**
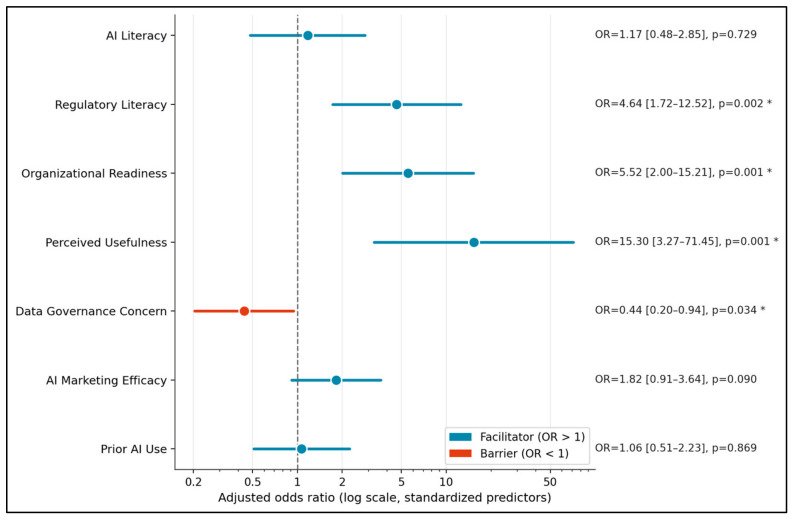
Forest plot of standardized adjusted odds ratios with 95% confidence intervals from the age-adjusted exploratory logistic regression model; * = statistically significant.

Figure 3 displays the forest plot of standardized adjusted odds ratios from the exploratory logistic model. Perceived usefulness, organizational readiness, and regulatory literacy had positive associations with top-tertile AAI, whereas data governance concern had a negative association. The large odds ratios should be interpreted cautiously because the sample was modest and the outcome was a dichotomized version of the primary continuous attitude scale.

## 4. Discussion

### 4.1. Analysis of Findings

This cross-sectional survey of 127 Romanian medical and pharmaceutical professionals identified several associated factors for AI adoption intention among non-clinical healthcare and pharmaceutical roles. Pharmaceutical marketing professionals reported higher AAI than hospital administrators and pharmacy managers, but these differences should be interpreted as associations within a convenience sample rather than as causal effects of professional group. The age imbalance across groups, differences in prior AI exposure, and sector-specific organizational environments mean that group contrasts probably reflect a combination of workforce demographics, workflow incentives, compliance exposure, and institutional readiness.

The findings are broadly consistent with TAM and UTAUT expectations, in which perceived usefulness and ease-related judgments are central correlates of adoption intention. However, the high R^2^ and high AUC also indicate that the survey constructs are closely related within the same measurement occasion. Therefore, these results strengthen construct coherence but do not prove that changing usefulness perceptions or organizational readiness would necessarily produce subsequent implementation behavior.

The non-clinical focus adds value because AI adoption in administrative, pharmacy–management, and pharmaceutical–commercial settings is shaped by procurement, budget allocation, information governance, promotional compliance, and workflow redesign. Unlike clinicians, who often evaluate AI through diagnostic accuracy, liability, and patient-facing trust, these professionals may weigh return on investment, data-processing risk, internal policy approval, and the feasibility of embedding AI into routine organizational processes.

The moderation result should be interpreted cautiously. Higher regulatory literacy attenuated the positive association between AI literacy and adoption intention, suggesting that regulatory knowledge may add a more critical appraisal of AI deployment. This does not mean that regulatory literacy reduces adoption; rather, it may shift enthusiasm toward more selective, compliant, and institutionally governed use.

Implementation recommendations should therefore be balanced and segment-specific. Training and communication may help, but workforce attitudes alone are unlikely to determine adoption. Organizational infrastructure, interoperable data systems, cybersecurity maturity, procurement capacity, reimbursement incentives, financial resources, internal governance, and institutional policy constraints may all influence whether favorable adoption intention becomes real-world AI implementation. The exploratory clusters can guide hypothesis generation, but they should not be used as fixed labels for professional groups without replication.

### 4.2. Study Limitations

Several limitations qualify the interpretation of these findings. First, the cross-sectional design precludes causal inference and temporal ordering; indirect-effect, moderation, logistic, and ROC analyses should be understood as statistical associations within one survey occasion, not as evidence that one construct causes another or predicts future behavior. Second, the sample was modest and recruited through purposive convenience channels in Western Romania, so non-response bias and selection of AI-interested professionals are possible. Third, the Romanian questionnaire combined adapted scales with study-specific role-adapted items and custom literacy quizzes; although internal reliability, item difficulty, and discrimination were acceptable for exploratory analysis, formal external validation in a Romanian professional population remains necessary. Fourth, the top-tertile high-adopter outcome dichotomized a continuous attitude scale and was used only as a supplementary convergence analysis; it may reduce information and should not replace the primary continuous AAI model. Fifth, clustering was exploratory and requires replication in larger multi-site cohorts. Finally, the study measured intention and budget willingness rather than observed AI procurement, deployment, sustained use, or patient/organizational outcomes.

## 5. Conclusions

In this cross-sectional Romanian sample, AI adoption intention among hospital administrators, pharmaceutical marketing professionals, and community pharmacy managers was most consistently associated with perceived usefulness and organizational readiness, while data governance concern showed a negative association. Pharmaceutical marketing professionals reported the highest adoption intention, but this difference should be viewed in the context of younger age, higher prior AI use, and distinct commercial workflows rather than as a causal effect of professional category.

Future research should use longitudinal, multi-site designs that follow professionals and organizations from stated intention to actual AI procurement, implementation, governance review, and sustained use. External validation of the OLS, clustering, and logistic models in independent Romanian and international cohorts is needed before the findings can be translated into workforce screening, policy, or implementation decisions.

## Figures and Tables

**Table 1:** Sample characteristics by professional group.

Variable	Total (N = 127)	Hospital Admin (n = 43)	Pharma Marketing (n = 42)	Pharmacy Mgr (n = 42)	*p*-Value
Age (years), mean ± SD	40.2 ± 8.2	42.3 ± 9.0	36.7 ± 6.7	41.7 ± 7.5	0.002
Female, n (%)	79 (62.2%)	27 (62.8%)	26 (61.9%)	26 (61.9%)	0.995
Experience (years), mean ± SD	12.9 ± 7.6	16.8 ± 7.9	8.8 ± 5.3	13.1 ± 7.1	<0.001
MBA/mgmt. degree, n (%)	54 (42.5%)	18 (41.9%)	22 (52.4%)	14 (33.3%)	0.209
Prior professional AI use, n (%)	70 (55.1%)	21 (48.8%)	32 (76.2%)	17 (40.5%)	0.003
AI Literacy (0–10), mean ± SD	6.1 ± 1.8	5.9 ± 1.5	7.2 ± 1.2	5.1 ± 2.0	<0.001
Regulatory Literacy (0–10), mean ± SD	6.1 ± 1.7	6.1 ± 1.4	6.8 ± 1.8	5.5 ± 1.8	0.002
Organizational Readiness (1–5), mean ± SD	3.4 ± 0.7	3.4 ± 0.6	3.7 ± 0.5	3.0 ± 0.7	<0.001
Budget Allocation Willingness (%), mean ± SD	14.9 ± 5.8	14.6 ± 4.0	20.6 ± 3.5	9.7 ± 3.5	<0.001

**Table 2:** Adoption-relevant outcomes by professional group.

Outcome (Scale)	Total	Hospital Admin	Pharma Marketing	Pharmacy Mgr	F (df = 2124)	*p*-Value	η^2^
AI Adoption Intention (1–5)	3.53 ± 0.78	3.39 ± 0.47	4.33 ± 0.50	2.88 ± 0.54	88.42	<0.001	0.588
Perceived Usefulness (1–5)	3.70 ± 0.73	3.57 ± 0.65	4.29 ± 0.49	3.23 ± 0.59	36.65	<0.001	0.372
Perceived Ease of Use (1–5)	3.32 ± 0.72	3.29 ± 0.67	3.72 ± 0.63	2.95 ± 0.66	14.45	<0.001	0.189
Organizational Readiness (1–5)	3.36 ± 0.69	3.40 ± 0.58	3.72 ± 0.53	2.96 ± 0.74	15.84	<0.001	0.204
Data Governance Concern (1–5)	3.76 ± 0.70	4.10 ± 0.49	3.40 ± 0.74	3.77 ± 0.70	12.27	<0.001	0.165
AI Marketing Efficacy (1–5)	3.42 ± 0.73	3.23 ± 0.68	3.94 ± 0.45	3.08 ± 0.72	22.36	<0.001	0.265
Budget Allocation Willingness (%)	14.9 ± 5.8	14.6 ± 4.0	20.6 ± 3.5	9.7 ± 3.5	93.09	<0.001	0.600
AI use cases considered (0–8)	4.03 ± 1.97	4.00 ± 2.01	4.69 ± 1.99	3.40 ± 1.73	4.74	0.010	0.071

**Table 3:** Tukey HSD pairwise comparisons for AI adoption intention.

Comparison	Mean Difference	95% CI (Lower)	95% CI (Upper)	*p*-Adj	Cohen’s d
Pharma Marketing vs. Hospital Admin	0.94	0.68	1.20	<0.001	1.94
Hospital Admin vs. Pharmacy Mgr	0.51	0.25	0.77	<0.001	1.00
Pharma Marketing vs. Pharmacy Mgr	1.45	1.19	1.71	<0.001	2.76

**Table 4:** Spearman’s rank correlations among key study variables.

Variable	AAI	AI Lit	Reg Lit	OR	PU	PEOU	DGC	AME	BAW
AI Adoption Intention (AAI)	1.00	0.65 ***	0.40 ***	0.48 ***	0.74 ***	0.56 ***	−0.29 ***	0.46 ***	0.73 ***
AI Literacy	0.65 ***	1.00	0.17	0.17	0.66 ***	0.52 ***	−0.17	0.19 *	0.62 ***
Regulatory Literacy	0.40 ***	0.17	1.00	0.17	0.28 **	0.13	−0.06	0.07	0.23 **
Organizational Readiness	0.48 ***	0.17	0.17	1.00	0.26 **	0.17	−0.15	0.22 *	0.44 ***
Perceived Usefulness (PU)	0.74 ***	0.66 ***	0.28 **	0.26 **	1.00	0.42 ***	−0.17	0.26 **	0.70 ***
Perceived Ease of Use	0.56 ***	0.52 ***	0.13	0.17	0.42 ***	1.00	−0.08	0.23 **	0.42 ***
Data Governance Concern	−0.29 ***	−0.17	−0.06	−0.15	−0.17	−0.08	1.00	−0.20 *	−0.12
AI Marketing Efficacy	0.46 ***	0.19 *	0.07	0.22 *	0.26 **	0.23 **	−0.20 *	1.00	0.39 ***
Budget Allocation Willingness	0.73 ***	0.62 ***	0.23 **	0.44 ***	0.70 ***	0.42 ***	−0.12	0.39 ***	1.00

* *p* < 0.05; ** *p* < 0.01; *** *p* < 0.001.

**Table 5:** Age-adjusted multivariable OLS regression predicting AI adoption intention with HC3 robust standard errors.

Predictor	β	HC3 SE	z	95% CI	*p*-Value
Constant	0.061	0.348	0.17	[−0.621, 0.742]	0.861
AI Literacy (0–10)	0.067	0.025	2.67	[0.018, 0.116]	0.008
Regulatory Literacy (0–10)	0.078	0.023	3.35	[0.032, 0.124]	0.001
Organizational Readiness (1–5)	0.206	0.056	3.67	[0.096, 0.317]	<0.001
Perceived Usefulness (1–5)	0.318	0.071	4.49	[0.179, 0.456]	<0.001
Perceived Ease of Use (1–5)	0.162	0.056	2.89	[0.052, 0.273]	0.004
Data Governance Concern (1–5)	−0.117	0.050	−2.36	[−0.215, −0.020]	0.018
AI Marketing Efficacy (1–5)	0.139	0.052	2.66	[0.037, 0.242]	0.008
Prior professional AI use (1/0)	0.018	0.075	0.24	[−0.129, 0.164]	0.812
MBA/management degree (1/0)	−0.146	0.067	−2.18	[−0.278, −0.014]	0.030
Experience (years)	−0.001	0.004	−0.27	[−0.010, 0.007]	0.784
Age (years)	−0.006	0.004	−1.48	[−0.014, 0.002]	0.142
Female (1/0)	0.010	0.071	0.14	[−0.129, 0.149]	0.890
Hospital Admin (vs. Pharmacy)	0.182	0.090	2.02	[0.006, 0.358]	0.043
Pharma Marketing (vs. Pharmacy)	0.436	0.124	3.51	[0.192, 0.680]	<0.001

R^2^ = 0.833; Adjusted R^2^ = 0.811; F(14, 112) = 39.72, *p* < 0.001. Maximum variance-inflation factor = 3.94.

**Table 6:** Exploratory k-means cluster profiles based on AAI, AI literacy, organizational readiness, perceived usefulness, data governance concern, and AI marketing efficacy.

Feature	Cluster 1: Governance-Cautious Segment (n = 35)	Cluster 2: Low-Readiness Segment (n = 45)	Cluster 3: High-Readiness Segment (n = 47)
AI Adoption Intention	3.41 ± 0.42	2.83 ± 0.48	4.30 ± 0.49
AI Literacy (0–10)	6.75 ± 1.08	4.28 ± 1.28	7.27 ± 1.34
Organizational Readiness	3.22 ± 0.56	3.13 ± 0.73	3.69 ± 0.63
Perceived Usefulness	3.86 ± 0.44	2.99 ± 0.48	4.26 ± 0.49
Data Governance Concern	4.17 ± 0.59	3.88 ± 0.59	3.34 ± 0.66
AI Marketing Efficacy	2.80 ± 0.61	3.27 ± 0.60	4.02 ± 0.40
Budget Allocation Willingness (%)	14.9 ± 4.4	10.3 ± 3.8	19.4 ± 4.6
Hospital Administrators, n	22 (62.9%)	17 (37.8%)	4 (8.5%)
Pharmaceutical Marketing, n	4 (11.4%)	0 (0.0%)	38 (80.9%)
Community Pharmacy Managers, n	9 (25.7%)	28 (62.2%)	5 (10.6%)

Cluster-group association: chi-square (4) = 89.39, *p* = 1.78 × 10^−18^. Cluster number selection was supported by the elbow method and the highest mean silhouette score for k = 3 (0.46).

**Table 7:** Bootstrap indirect-effect analyses through perceived usefulness.

Model	Path a (X to M)	Path b (M to Y|X)	Total Association (c)	Direct Association (c’)	Indirect Association (a × b) [95% CI]	*p*	% of Association Accounted for
AI Literacy → PU → AAI	0.265	0.629	0.270	0.104	0.167 [0.119, 0.221]	<0.001	61.7%
Org. Readiness → PU → AAI	0.273	0.774	0.567	0.356	0.211 [0.086, 0.326]	<0.001	37.3%
DGC → PU → AAI	−0.176	0.869	−0.330	−0.177	−0.153 [−0.279, −0.018]	0.024	46.4%

X = predictor; M = perceived usefulness; Y = AI adoption intention. Estimates represent indirect statistical associations, not causal mediation.

**Table 8:** Moderation analysis: Regulatory literacy moderates the AI literacy/AAI relationship.

Term	β	HC3 SE	z	95% CI	*p*-Value
Constant	3.081	0.071	43.33	[2.942, 3.221]	<0.001
AI Literacy (centered)	0.139	0.021	6.51	[0.097, 0.181]	<0.001
Regulatory Literacy (centered)	0.103	0.024	4.24	[0.055, 0.150]	<0.001
AI Literacy × Regulatory Literacy	−0.027	0.013	−2.02	[−0.053, −0.001]	0.043
Data Governance Concern (centered)	−0.096	0.072	−1.33	[−0.237, 0.045]	0.182
AI Literacy × DGC	−0.031	0.033	−0.94	[−0.096, 0.034]	0.346
Hospital Admin (vs. Pharmacy)	0.380	0.097	3.93	[0.190, 0.569]	<0.001
Pharma Marketing (vs. Pharmacy)	0.995	0.112	8.86	[0.774, 1.215]	<0.001
Simple slopes of AI Literacy at:					
Low Regulatory Literacy (−1 SD)	0.186	0.029	6.46	—	<0.001
Mean Regulatory Literacy	0.139	0.021	6.51	—	<0.001
High Regulatory Literacy (+1 SD)	0.093	0.034	2.76	—	0.007

Model R^2^ = 0.736; Adjusted R^2^ = 0.721; F(7, 119) = 54.20, *p* < 0.001.

**Table 9:** Age-adjusted exploratory logistic regression and ROC discrimination for high-adopter status.

Predictor	β	SE	Odds Ratio	95% CI	*p*-Value
Constant	−2.196	0.543	0.11	[0.04, 0.32]	<0.001
AI Literacy (z-score)	0.157	0.453	1.17	[0.48, 2.85]	0.729
Regulatory Literacy (z-score)	1.536	0.506	4.64	[1.72, 12.52]	0.002
Organizational Readiness (z-score)	1.708	0.517	5.52	[2.00, 15.21]	0.001
Perceived Usefulness (z-score)	2.728	0.786	15.30	[3.27, 71.45]	<0.001
Data Governance Concern (z-score)	−0.824	0.389	0.44	[0.20, 0.94]	0.034
AI Marketing Efficacy (z-score)	0.599	0.353	1.82	[0.91, 3.64]	0.090
Prior professional AI use (1/0)	0.062	0.378	1.06	[0.51, 2.24]	0.869
Age (z-score)	−0.117	0.229	0.89	[0.57, 1.39]	0.609

Pseudo-R^2^ = 0.660; likelihood-ratio test *p* = 3.98 × 10^−20^; ROC AUC = 0.966 (bootstrap 95% CI 0.934-0.988); Youden-optimal cut-off sensitivity = 0.86, specificity = 0.96. Classification was exploratory and internally estimated.

## Data Availability

The data presented in this study are available on request from the corresponding author. The data are not publicly available due to privacy and ethical restrictions.

## References

[1] Topol E.J. (2019). High-performance medicine: The convergence of human and artificial intelligence. Nat. Med..

[2] World Health Organization (2021). Global Strategy on Digital Health 2020–2025.

[3] Davenport T., Kalakota R. (2019). The potential for artificial intelligence in healthcare. Future Healthc. J..

[4] Debnath B., Wilson S.K., Basu S., Kompella S.Y., Singha R., Sahoo S.K., Yadav N., Roy P., Kundu A. (2025). The Pharmaceutical Industry’s Future: How Artificial Intelligence is Transforming Medicine. Adv. Pharm. Bull..

[5] Deloitte (2024). 2024 Global Life Sciences Sector Outlook: Realizing Generative AI Value in Life Sciences.

[6] Alhashmi S.F.S., Salloum S.A., Abdallah S. (2020). Critical success factors for implementing artificial intelligence (AI) projects in Dubai government United Arab Emirates (UAE) healthcare sector: Applying the extended technology acceptance model (TAM). International Conference on Advanced Intelligent Systems and Informatics.

[7] Davis F.D. (1989). Perceived usefulness, perceived ease of use, and user acceptance of information technology. MIS Q..

[8] Venkatesh V., Morris M.G., Davis G.B., Davis F.D. (2003). User acceptance of information technology: Toward a unified view. MIS Q..

[9] Weiner B.J. (2009). A theory of organizational readiness for change. Implement. Sci..

[10] Rogers E.M. (2003). Diffusion of Innovations.

[11] Gao S., He L., Chen Y., Li D., Lai K. (2020). Public perception of artificial intelligence in medical care: Content analysis of social media. J. Med. Internet Res..

[12] European Union (2016). Regulation (EU) 2016/679 of the European Parliament and of the Council of 27 April 2016 (General Data Protection Regulation). Off. J. Eur. Union.

[13] European Union (2017). Regulation (EU) 2017/745 of the European Parliament and of the Council of 5 April 2017 on Medical Devices. Off. J. Eur. Union.

[14] European Union (2024). Regulation (EU) 2024/1689 of the European Parliament and of the Council of 13 June 2024 Laying Down Harmonised Rules on Artificial Intelligence (Artificial Intelligence Act). Off. J. Eur. Union.

[15] Ng D.T.K., Leung J.K.L., Chu S.K.W., Qiao M.S. (2021). Conceptualizing AI literacy: An exploratory review. Comput. Educ. Artif. Intell..

[16] Vladescu C., Scintee S.G., Olsavszky V., Hernandez-Quevedo C., Sagan A. (2016). Romania: Health System Review.

[17] Păcuraru I.M., Năstac A., Zamfir A., Busnatu Ș.S., Andronic O., Artamonov A.R. (2025). Digital Transformation of Medical Services in Romania: Does the Healthcare System Meet the Current Needs of Patients?. Healthcare.

[18] Levai C.M., Nussbaum L.A., Cojocaru A., Popa D.I., Tomescu A.M., Jugănaru I. (2026). Health Literacy and Acceptance of AI/XR-Enabled Telemedicine Among Romanian Medical Students: A Cross-Sectional Survey. Healthcare.

[19] Coman M.A., Forray A.I., Van den Broucke S., Chereches R.M. (2022). Measuring Health Literacy in Romania: Validation of the HLS-EU-Q16 Survey Questionnaire. Int. J. Public Health.

[20] Vandenbroucke J.P., von Elm E., Altman D.G., Gøtzsche P.C., Mulrow C.D., Pocock S.J., Poole C., Schlesselman J.J., Egger M., Strobe Initiative (2007). Strengthening the Reporting of Observational Studies in Epidemiology (STROBE): Explanation and elaboration. Epidemiology.

[21] Asan O., Bayrak A.E., Choudhury A. (2020). Artificial intelligence and human trust in healthcare: Focus on clinicians. J. Med. Internet Res..

[22] Sahoo R.K., Sahoo K.C., Negi S., Baliarsingh S.K., Panda B., Pati S. (2025). Health professionals’ perspectives on the use of Artificial Intelligence in healthcare: A systematic review. Patient Educ. Couns..

[23] McKinsey & Company (2024). Generative AI in the Pharmaceutical Industry: Moving from Hype to Reality.

[24] Hosmer D.W., Lemeshow S., Sturdivant R.X. (2013). Applied Logistic Regression.

[25] Longoni C., Bonezzi A., Morewedge C.K. (2019). Resistance to medical artificial intelligence. J. Consum. Res..

[26] Fishbein M., Ajzen I. (2010). Predicting and Changing Behavior: The Reasoned Action Approach.

[27] Prakash A.V., Das S. (2021). Medical practitioner’s adoption of intelligent clinical diagnostic decision support systems: A mixed-methods study. Inf. Manag..

[28] Sun T.Q., Medaglia R. (2019). Mapping the challenges of Artificial Intelligence in the public sector: Evidence from public healthcare. Gov. Inf. Q..

[29] Hayes A.F. (2022). Introduction to Mediation, Moderation, and Conditional Process Analysis: A Regression-Based Approach.

[30] Kaplan B. (2001). Evaluating informatics applications—Clinical decision support systems literature review. Int. J. Med. Inform..

